# Supplementary material for: Effector CD4+ T cells recognize intravascular antigen presented by patrolling monocytes
Source: Nat Commun. 2018 Feb 21;9:747. doi: 10.1038/s41467-018-03181-4 (PMC5821889; doi:10.1038/s41467-018-03181-4)
Supplement: Supplementary file 1 — Supplementary Information [file 41467_2018_3181_MOESM1_ESM.pdf]

Supplementary Information

Effector CD4$^+$ T cells recognize intravascular antigen presented by patrolling monocytes

Westhorpe *et al*.

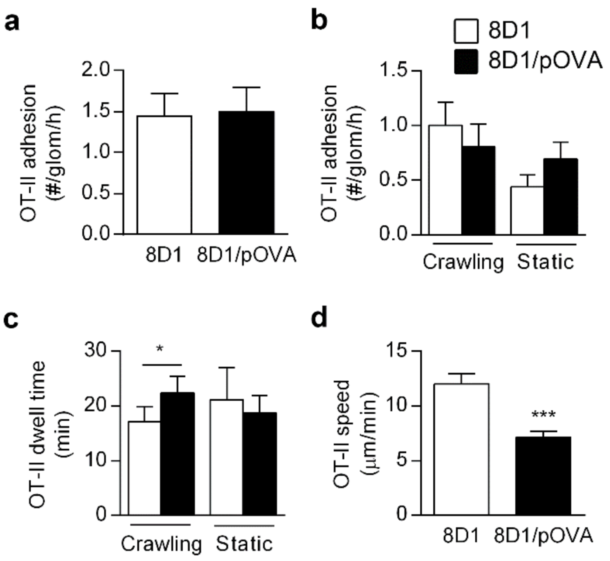

Supplementary Figure 1: Retention of OT-II cells in the glomeruli remains increased 2 hours after co-transfer with 8D1/pOVA. CFSE-labelled OT-II cells were transferred i.v. together with 150 µg of either 8D1 (white bars) or 8D1/pOVA (black bars). Intraglomerular retention and migration of OT-II cells were examined via intravital multiphoton microscopy 2-3 h after transfer and migration parameters were assessed. Data were derived from n=3 mice per group. Data are shown for the number of adherent OT-II cells (**a**), the number of crawling and static cells (**b**), dwell time for crawling and static OT-II cells (**c**) and migration speed of crawling OT-II cells (**d**). Data are presented as mean ± sem. In **c**, 8D1 - n=62 cells total, n=19 static & n=43 crawling; 8D1/pOVA - n=69 cells total, n=32 static & n=37 crawling. In **d**, 8D1 - n=27 cells; 8D1/pOVA - n=16 cells. *, $P < 0.05$; ***, $P < 0.001$ vs 8D1 via Mann-Whitney tests.

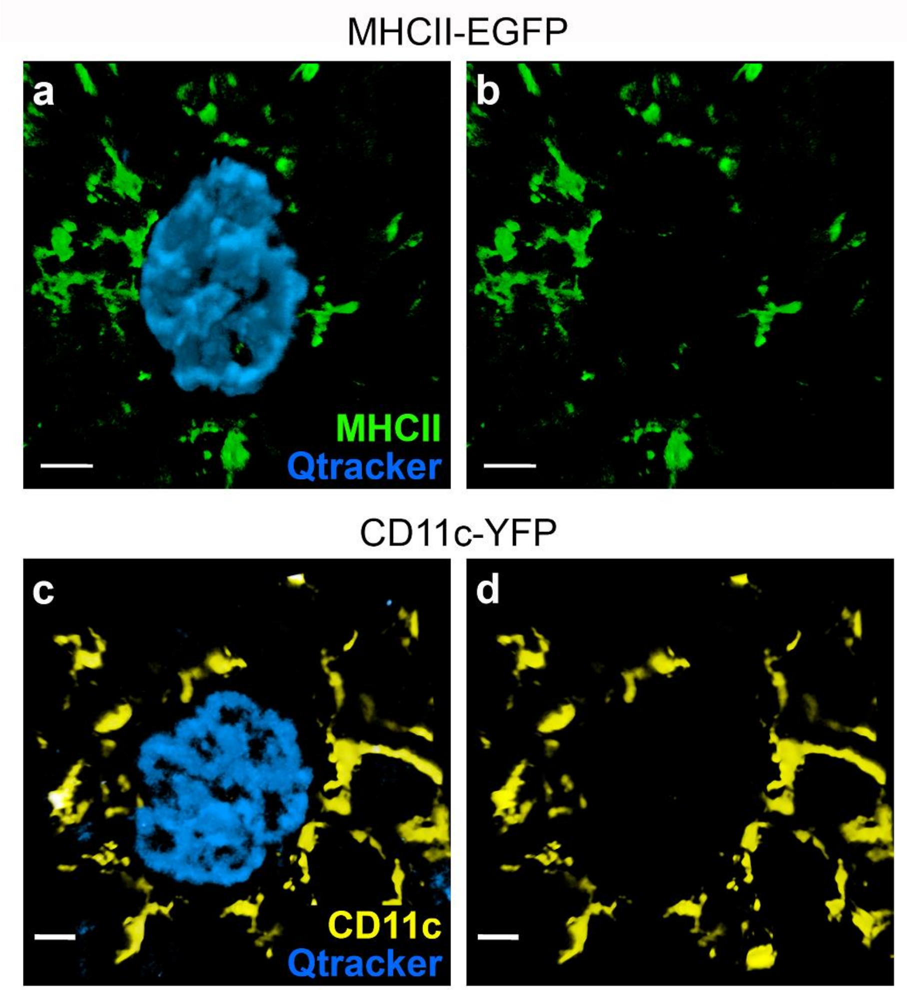

Supplementary Figure 2: MHCII-expressing renal interstitial dendritic cells are excluded from the glomerulus. Kidneys from MHCII-EGFP mice (**a, b**) and CD11c-YFP mice (**c, d**) were fixed and slices examined by multiphoton microscopy. The renal microvasculature was pre-labelled by i.v. administration of Qtracker®-655 (blue) prior to kidney removal. Images are maximum projections of glomerular regions in which the vascular signal is retained (**a, c**) or excluded (**b, d**). In MHCII-GFP mice (**a, b**), MHCII-expressing cells are visible via GFP (green). In CD11c-YFP mice (**c, d**), renal dendritic cells are visible via YFP expression (yellow). No projections from either cell type are visible in the glomerular space. Scale bars = 12 µm (**a, b**) and 15 µm (**c, d**). See also **Supplementary Movies 3** & **4**.

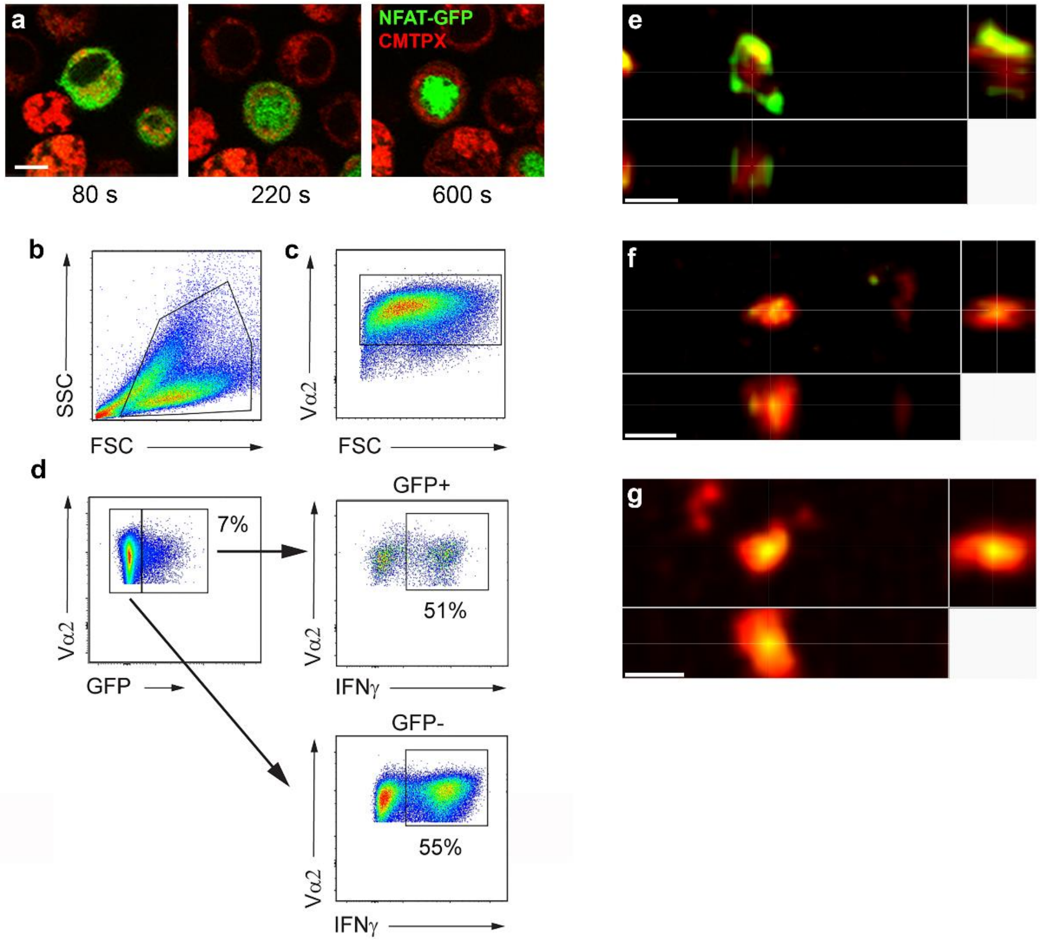

Supplementary Figure 3: Activated OT-II$_{NFAT\text{-}GFP}$ cells display nuclear NFAT-GFP translocation and IFNγ production. (**a**): OT-II$_{NFAT\text{-}GFP}$ cells were stained with CMTPX (red) and examined via live cell confocal microscopy following activation with PMA/ionomycin (time after activation shown below images). Images show the change in sub-cellular localization of NFAT-GFP (green) from cytoplasmic (80 s), to both nuclear and cytoplasmic (220 s), to exclusively nuclear (600 s). Scale bar = 5 μm. (**b-d**) Comparison of IFNγ production by NFAT-GFP-transduced OT-II cells and non-transduced OT-II cells from the same culture. OT-II cells were identified on the basis of forward and side scatter (**b**) and Vα2 expression (**c**). (**d**): Transduced and non-transduced OT-II cells, differentiated on the basis of GFP expression, were assessed for IFNγ production via intracellular staining following *in vitro* activation. The percentage of cells positive for IFNγ was comparable for both populations (representative data from n=2 experiments). (**e-g**) *In vivo* images of CMTPX-stained OT-II$_{NFAT\text{-}GFP}$ cells in the glomerulus, showing cells with cytoplasmic (**e**), nuclear and cytoplasmic (**f**) and exclusively nuclear NFAT-GFP (**g**), viewed as maximum projections in each of the *z*, *x* and *y* planes. Scale bars = 10 μm.

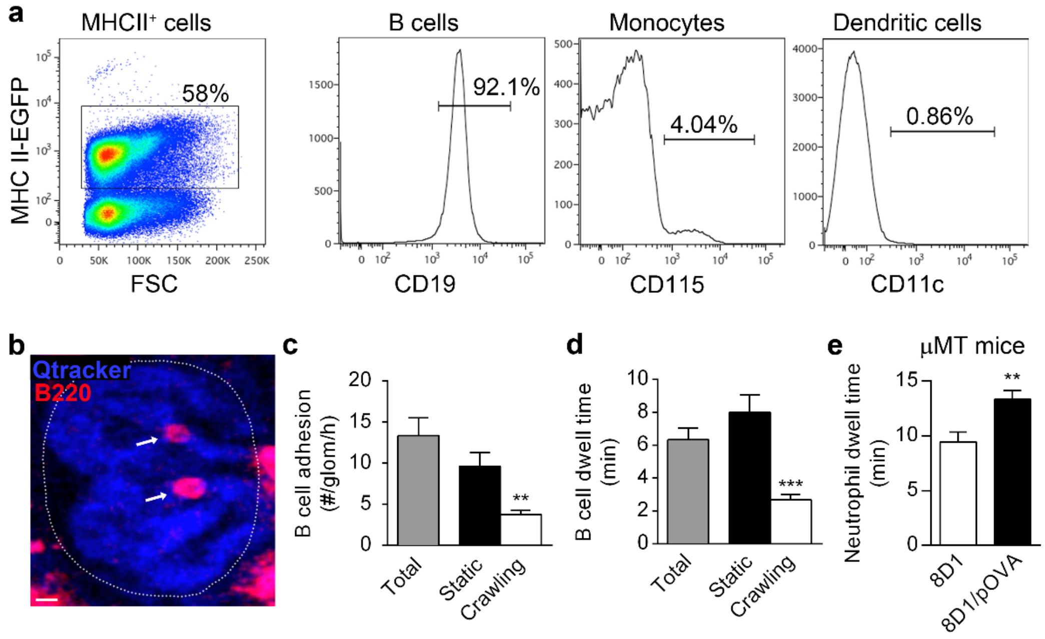

Supplementary Figure 4: B cells comprise the majority of circulating MHCII$^+$ leukocytes but are not required for antigen-dependent neutrophil recruitment induced by OT-II cells. (**a**): Blood from MHCII-EGFP mice was analyzed by flow cytometry for different antigen-presenting cell populations. MHCII-EGFP$^+$ cells (left panel) were identified in whole blood and expression of CD19, CD115 and CD11c determined to identify B cells, monocytes and dendritic cells respectively. Gates were set to 0.1% on fluorescence-minus-one control samples. Representative histograms are shown for each cell type, and for monocytes are representative of analyses from three mice. (**b-d**): Intravital multiphoton microscopy analysis of B cell adhesion and migration in glomerular capillaries. B cells were visualized using PE-conjugated anti-B220. (**b**): Representative image showing B cells (red, indicated by arrows) adherent in glomerular microvasculature (blue). See also **Supplementary Movie 9**. Scale bar represents 10 μm. (**c, d**): Number (**c**) and dwell time (**d**) of adherent B cells (data were derived from n=8 mice and are shown as mean ± sem). **, P < 0.01; ***, P < 0.001 vs 'static' via unpaired Student's *t*-tests with Welch's correction. In **d**, data are expressed per cell (n=186 total, 130 static & 56 crawling). (**e**): OT-II cell-induced neutrophil recruitment to the glomerulus was determined in B cell-deficient μMT mice. OT-II cells were transferred together with either 8D1 or 8D1/pOVA and neutrophil dwell time in the glomerular capillaries was measured by multiphoton microscopy after 4 h (n=5 mice per group). Data are shown as mean ± sem. **, P < 0.01 vs 8D1 via unpaired Student's *t*-test.

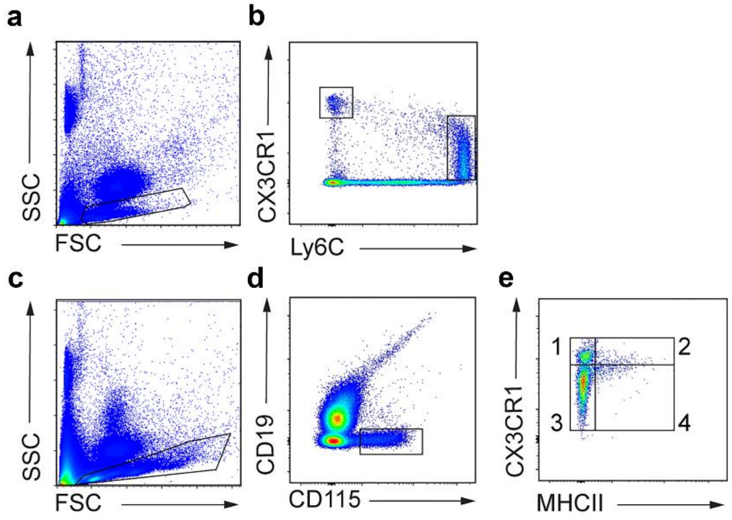

f

1.CX3CR1^{hi}	16.9 ± 4.4%
2.CX3CR1^{hi} MHCII^{+}	3.2 ± 0.4%
3.CX3CR1$^{lo/int}$	73.2 ± 2.3%
4.CX3CR1$^{lo/int}$ MHCII^{+}	6.3 ± 1.4%

Supplementary Figure 5: Expression of MHCII by classical and non-classical monocytes. (**a**, **b**) Single mononuclear blood leukocytes were identified from whole blood of *Cx3Cr1*$^{gfp/+}$ mice on the basis of forward and side scatter (**a**) and assessed for expression of CX3CR1 (GFP) and Ly6C (**b**), identifying distinct CX3CR1^{hi} Ly6C^{-ve} (non-classical) and CX3CR1$^{lo\text{-}int}$ Ly6C^{+} (classical) monocyte populations. (**c-e**) To assess MHCII expression by classical and non-classical monocytes, blood mononuclear cells were defined on the basis of forward and side scatter (**c**) and monocytes identified via CD115 expression, using CD19 expression to exclude B cells (**d**). (**e**): CD115^{+} CD19^{-} monocytes were then assessed for expression of CX3CR1 and MHCII. (**f**): % of monocytes in each of the following gates in (**e**): CX3CR1^{hi} MHCII^{-ve}, CX3CR1^{hi} MHCII^{+}, CX3CR1$^{lo\text{-}int}$ MHCII^{-ve}, and CX3CR1$^{lo\text{-}int}$ MHCII^{+}. Flow cytometry data shown are representative of n=3 mice for each staining strategy. Numerical data show mean ± sem of MHCII^{+} cells as % of total CD115^{+} CD19^{-} cells.

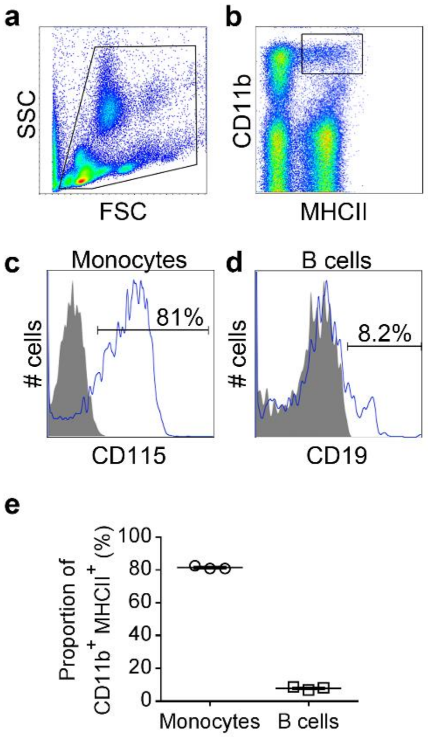

Supplementary Figure 6: Monocytes comprise the majority of CD11b$^+$ MHCII$^+$ cells in mouse blood. Flow cytometric analysis of blood from MHCII-EGFP mice. (**a, b**): Circulating CD11b$^+$ MHCII-EGFP$^+$ leukocytes were identified on the basis of forward and side scatter (**a**) and expression of CD11b and MHCII-EGFP$^+$ (**b** - gated population). (**c, d**): The proportions of monocytes and B cells (blue lines) were determined by staining with CD115 (**c**) and CD19 (**d**) respectively. Percentages are based on comparison with fluorescence-minus-one control staining (shaded histograms). Representative data shown. (**e**): Average data (mean ± sem, with individual data points shown), derived from n=3 mice.

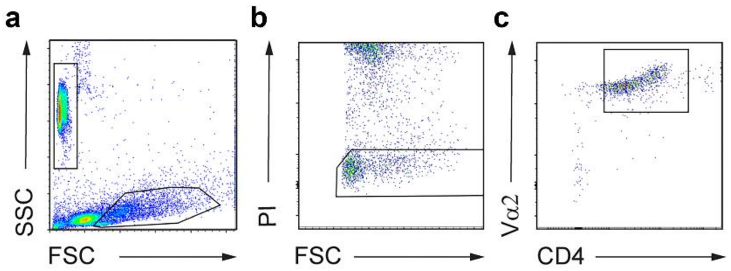

Supplementary Figure 7: Gating for identification of OT-II cells in *in vitro* proliferation assays. (**a-c**): OT-II cells were identified from cultures on the basis of forward scatter/side scatter (**a** – polygonal gate), viability, based on propidium iodide exclusion (**b**), and high expression of Vα2 and CD4 (**c**). In (**a**), a high SSC rectangular gate used to define counting beads is also shown.

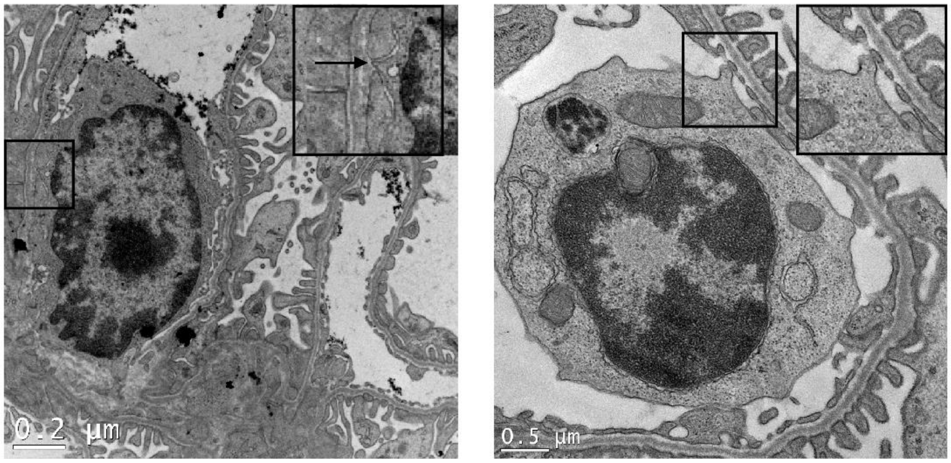

Supplementary Figure 8: **Electron microscopic assessment of immune cell probing adjacent to endothelial fenestrations in glomerular capillaries.** Otherwise untreated kidneys were fixed and prepared for electron microscopy. Images show mononuclear leukocytes within glomerular capillaries, closely apposed to the endothelial surface. Regions within insets (magnified in the top right corner) show leukocyte microvilli within (arrow) or adjacent to fenestrations. Scale bars indicate either 0.2 or 0.5 μm.

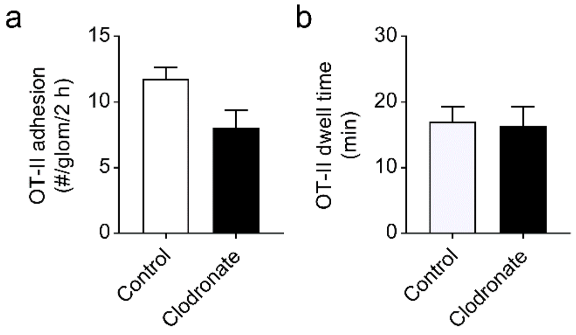

Supplementary Figure 9: Effect of clodronate on glomerular effector T cell recruitment and migration. Mice were treated with either control or clodronate-loaded liposomes and subsequently injected with 1 x 10^7 activated OT-II T cells. T cell adhesion in glomeruli was assessed by multiphoton microscopy in the 2 hours following injection. Data are shown for number (**a**) and dwell time (**b**) of adherent OT-II cells. Data are shown as mean ± sem of n=5 mice per group.

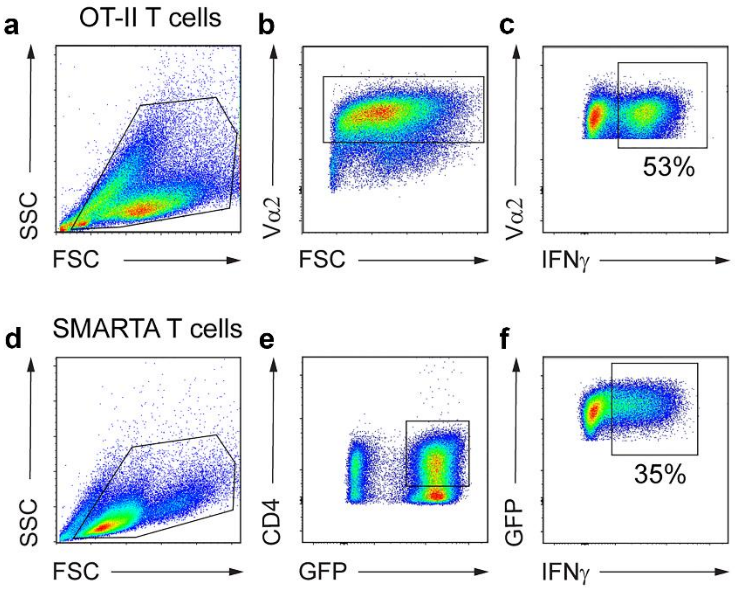

Supplementary Figure 10: SMARTA T cells undergo antigen-dependent activation *in vitro* in a manner similar to OT-II cells. (**a-f**): OT-II T cells and SMARTA T cells (from SMARTA-GFP mice) underwent antigen-induced activation *in vitro*, using the OVA and LCMV peptides respectively. To assess activation, at the end of the 7 day activation protocol, T cells were restimulated with PMA/ionomycin in the presence of Brefeldin A and stained for intracellular IFNγ (**c, f**). (**a-c**): OT-II cells were identified on the basis of forward and side scatter (**a**) and Vα2 expression (**b**), and expression of IFNγ determined (**c**). (**d-e**): SMARTA cells were identified on the basis of forward and side scatter (**d**) and expression of CD4 and GFP (**e**), and expression of IFNγ determined (**f**). The numbers below the gates in **c** & **f** indicate the proportion of cells positive for IFNγ in each culture, relative to the relevant isotype control.

Supplementary Table 1: Antibodies used for flow cytometry cell culture and *in vivo* imaging

Target	Clone	Supplier	Used in
8D1	8D1	In-house	[15, 33]
Control IgG	MOPC-21	BioXCell	[58]
IL-4	11B11	ATCC	[15]
CD4	GK1.5	BD Pharmingen	[59]
B220	RA3-6B2	BD Pharmingen	[60]
CD11b	M1/70	eBioscience	[3]
Gr-1	RB6-8C5	eBioscience	[17]
CD115	AFS98	eBioscience	[61]
CD45	30-F11	BD Pharmingen	[39, 40]
CD19	1D3	BD Pharmingen	[39, 40]
CD11c	HL3	BD Pharmingen	[39, 40]
Ly6C	AL-21	BD Pharmingen	[39, 40]
IFNγ	XMG1.2	BD Pharmingen	[62]
TCR Vβ5.1/5.2	MR9-4	BD Pharmingen	[62]
TCR Vα2	B20.1	In-house	[39, 40]
MHC Class II	M5/114.15.2	BD Pharmingen	[39, 40]
