# Supplementary material for: Effector CD4+ T cells recognize intravascular antigen presented by patrolling monocytes
Source: Nat Commun. 2018 Feb 21;9:747. doi: 10.1038/s41467-018-03181-4 (PMC5821889; doi:10.1038/s41467-018-03181-4)
Supplement: Supplementary file 3 — Descriptions of Additional Supplementary Files [file 41467_2018_3181_MOESM3_ESM.pdf]

Description of Additional Supplementary Files

File Name: Supplementary Movie 1
Description: **CD4$^+$ T cells undergo constitutive migration in glomerular capillaries.** *In vivo* multiphoton microscopy movie showing adhesion and migration by CD4$^+$ T cells in a non-inflamed C57BL/6 mouse. CD4$^+$ T cells are stained via anti-CD4 mAb (red), and the vasculature is labelled via Qtracker®-655 (blue). Circulating endogenous CD4$^+$ T cells can be seen undergoing predominantly short-lived interactions in glomerular capillaries. Real time duration – 16 min. Scale bar = 15 µm.

File Name: Supplementary Movie 2
Description: **Effector OT-II cells undergo migration in the glomerular capillaries in non-inflamed conditions.** *In vivo* multiphoton microscopy movie showing a CFSE-labelled OT-II cell (green) adhering and migrating in the glomerular capillaries of a non-inflamed C57BL/6 mouse. The vasculature is labelled via Qtracker®-655 (blue). Real time duration – 27.5 min. Scale bar = 10 µm.

File Name: Supplementary Movie 3
Description: **3 dimensional assessment of MHCII-expressing cells adjacent to glomeruli.** *Ex vivo* 3-dimensional multiphoton microscopy movie of a glomerulus in a MHCII-EGFP mouse. MHCII-expressing cells are visible via GFP (green) and the glomerular vasculature is labelled via Qtracker®655 (blue). The first portion of the movie shows a rotating view of a 3-dimensional reconstruction of the glomerulus, with and without the glomerular vasculature, showing the network of MHCII-expressing cells around the glomerulus. The second portion of the movie focusses through the glomerulus from top to bottom. Scale bar = 20 µm.

File Name: Supplementary Movie 4
Description: **3 dimensional assessment of CD11c-expressing cells adjacent to glomeruli.** *Ex vivo* 3-dimensional multiphoton microscopy movie of a glomerulus in a CD11c-YFP mouse. CD11c-expressing cells are visible via YFP (yellow) and the glomerular vasculature is labelled via Qtracker®655 (blue). The first portion of the movie shows a rotating view of a 3-dimensional reconstruction of the glomerulus, with and without the glomerular vasculature, showing the network of CD11c-expressing cells around the glomerulus. The second portion of the movie focusses through the glomerulus from top to bottom. Scale bar = 20 µm.

File Name: Supplementary Movie 5
Description: **MHCII-EGFP$^+$ leukocytes undergo constitutive migration in the glomerular capillaries.** *In vivo* multiphoton microscopy movie of the glomerular capillaries in post-UUO MHCII-EGFP mouse. Leukocytes expressing MHCII-EGFP (green) are visible undergoing mostly short-duration interactions in the glomerular capillaries, which are labelled via Qtracker-655 (blue), although occasional MHCII-EGFP$^+$ leukocytes adhere for longer periods. Real time duration – 42 min. Scale bar = 10 µm.

File Name: Supplementary Movie 6
Description: **MHCII-EGFP$^+$ leukocytes undergo constitutive migration in the glomerular capillaries in intact kidneys.** *In vivo* multiphoton microscopy movie of two adjacent glomeruli in the kidney of a 3-week old MHCII-EGFP mouse. Staining was performed as per Movie S3. MHCII-EGFP$^+$ leukocytes can be seen intermittently undergoing prolonged retention (arrows) in glomerular capillaries. Real time duration – 21.5 min. Scale bar = 20 µm.

File Name: Supplementary Movie 7
Description: **Effector OT-II cells interact with MHCII-EGFP^{+} leukocytes in the glomerular capillaries.** *In vivo* multiphoton microscopy movie of a CMTPX-labelled OT-II cell (red) interacting with an MHCII-EGFP^{+} leukocyte (green). The vasculature is labelled via Qtracker®-655 (blue). The interacting cells remain in close proximity for an extended period. Real time duration – 30.5 min.

File Name: Supplementary Movie 8
Description: **OT-II cells with cytoplasmic versus nuclear NFAT-GFP display different migratory function.** *In vivo* multiphoton microscopy movies of the glomerular capillaries of a C57BL/6 mice following administration of 8D1/pOVA and CMTPX-stained OT-II$_{NFAT\text{-}GFP}$ cells. In the left hand movie, OT-II$_{NFAT\text{-}GFP}$ cells with cytoplasmic NFAT-GFP (green) are seen displaying active, migratory behavior. In the right hand movie, an OT-II$_{NFAT\text{-}GFP}$ cell displaying nuclear NFAT-GFP remains static. Real time duration – 10 min. Scale bars = 10 µm.

File Name: Supplementary Movie 9
Description: **B cells undergo constitutive migration in the glomerular capillaries.** *In vivo* multiphoton microscopy movie of the glomerular capillaries of a non-inflamed C57BL/6 mouse. B cells are v via anti-B220 mAb (red), and the vasculature is labelled via Qtracker-655 (blue). B cells are visible undergoing short-term adhesion in the glomerular capillaries. Real time duration – 40 min. Scale bar = 15 µm.

File Name: Supplementary Movie 10
Description: **CD11b^{+} MHCII-EGFP^{+} leukocytes undergo prolonged retention in glomerular capillaries under non-inflamed conditions.** *In vivo* multiphoton microscopy movie of the glomerular capillaries of an uninflamed MHCII-EGFP mouse. MHCII-EGFP^{+} leukocytes are visualized via GFP (green) and CD11b^{+} leukocytes (predominantly monocytes and neutrophils) are visualized via anti-CD11b mAb (red). The vasculature is labelled via Qtracker-655 (blue). The left panel shows the merged movie, while in the right panel, the CD11b stain is omitted to clearly demonstrate MHCII-EGFP expression. Two CD11b^{+} MHCII-EGFP^{+} leukocytes (arrows) are seen crawling in a glomerulus for extended periods. In addition, numerous CD11b^{+} MHCII-EGFP^{-} leukocytes (red – predominantly monocytes and neutrophils) and CD11b^{-} MHCII-EGFP^{+} leukocytes (green – predominantly B cells) can be seen undergoing shorter periods of retention. Real time duration – 53 min. Scale bar = 10 µm.

File Name: Supplementary Movie 11
Description: **Neutrophils generate reactive oxygen species in the glomerulus following transfer of effector OT-II cells and 8D1/pOVA.** *In vivo* multiphoton microscopy movie of the glomerular capillaries of a C57BL/6 mouse treated with control liposomes, followed by administration of OT-II cells and planted antigen, and multiphoton imaging performed 24 h later. Neutrophils are labelled via anti-Gr-1 mAb (green), and generation of reactive oxygen species is detected via DHE staining (red). The vasculature is labelled via Qtracker-655 (grey). The left panel shows the merged movie, while in the right panel, the anti-Gr1 signal is omitted to clearly demonstrate DHE staining. A neutrophil is seen to become DHE-positive shortly after its recruitment to the glomerulus, indicating rapid induction of reactive oxygen species generation. DHE staining remains positive throughout the recording. Real time duration – 30 min. Scale bar = 15 µm.
